# Effects of Dietary Selenium and Oxidized Fish Oils on Intestinal Lipid Metabolism and Antioxidant Responses of Yellow Catfish *Pelteobagrus fulvidraco*

**DOI:** 10.3390/antiox11101904

**Published:** 2022-09-26

**Authors:** Guang-Hui Liu, Dian-Guang Zhang, Xi-Jun Lei, Xiao-Ying Tan, Chang-Chun Song, Hua Zheng, Zhi Luo

**Affiliations:** 1 Hubei Hongshan Laboratory, Fishery College, Huazhong Agricultural University, Wuhan 430070, China; 2 Laboratory for Marine Fisheries Science and Food Production Processes, Qingdao National Laboratory for Marine Science and Technology, Qingdao 266237, China

**Keywords:** selenium, selenoprotein, oxidized fish oils, oxidative stress, lipid metabolism, vertebrates

## Abstract

Currently, the effect of selenium and oxidized fish oil interactions on the intestinal lipid metabolism and antioxidant responses of fish remains unknown. Herein, yellow catfish *Pelteobagrus fulvidraco* (weight: 3.99 ± 0.01 g) were used as experimental animals and were fed four diets: an adequate amount of selenium (0.25 mg kg^−1^) with fresh fish oil (A-Se+FFO), an adequate amount of selenium with oxidized fish oil (A-Se+OFO), a high amount of selenium (0.50 mg kg^−1^) with fresh fish oil (H-Se+FFO), and a high amount of selenium with oxidized fish oil (H-Se+OFO). The feeding experiment was conducted for 10 weeks. The results showed that selenium supplementation alleviated the intestinal tissue damage and reduced the lipid accumulation that was induced by oxidized fish oils. Meanwhile, we also found that 0.50 mg kg^−1^ selenium reduced the oxidative stress that is caused by oxidized fish oils through increasing the GSH and the activity and mRNA expression of antioxidant enzymes. Dietary selenium and oxidized fish oils also affected the mRNA expression of intestinal selenoproteins including *selenow2a*, *selenop2*, and *selenot2*. Mechanistically, Se and oxidized eicosapentaenoic acid (oxEPA) influenced the GSH content by affecting the DNA binding ability of activating transcription factor (ATF) 3 to the *slc7a11* promoter. For the first time, our results suggested that selenium alleviated the oxidized fish oil-induced intestinal lipid deposition and the oxidative stress of the fish. We also elucidated the novel mechanism of selenium increasing the GSH content by affecting the interaction of ATF3 and the *slc7a11* promoter.

## 1. Introduction

Fish oils contain rich omega-3 long-chain polyunsaturated fatty acids (PUFA), such as eicosapentaenoic acid (EPA, 20:5n-3) and docosahexaenoic acid (DHA, 22:6n-3), which are beneficial for the health of humans and animals [1]. However, fish oils have multiple unsaturated bonds in the fatty acid chains, which makes them highly sensitive to oxidation when they are in ambient conditions [2]. In contrast to the advantages of fish oil, oxidized fish oils disrupt redox homeostasis and induces oxidative stress, and accordingly, they are harmful [3,4]. Studies have also suggested that dietary oxidized fish oils induced lipid deposition by enhancing lipogenesis and weakening lipolysis, as was found in loach (*Misgurnus anguillicaudatus*) [5], largemouth bass (*Micropterus salmoides*) [6], channel catfish (*Ictalurus punctatus*) [4], and yellow catfish (*Pelteobagrus fulvidraco*) [7]. The intestine is the main tissue for the digestion and uptake of nutrients [8]. The intestine is not a native organ for lipid deposition, and excessive lipid deposition in the intestine will adversely influence its function. However, research relevant with the assessment of the effect of oxidized fish oils on intestinal lipid deposition and metabolism are limited.

On the other hand, selenium is an essential micro-element for vertebrates, including fish [9,10]. Selenium performs its biological effects mainly through selenoproteins [11]. Nowadays, more than 50 selenoproteins have been identified, including the 24 and 25 selenoproteins that are in rodents and humans, respectively [12]. Recently, we reported 28 selenoproteins in yellow catfish [13]. Studies have suggested that selenium has a high antioxidant activity, and accordingly, it acts in the regulation of cellular redox homeostasis [9,14]. Other studies have reported that selenium and selenoproteins play essential roles in the control of lipid metabolism [7,15]. Thus, taking into account the significant role that is played by selenium in antioxidant capacity and lipid metabolism, we hypothesize that selenium could mitigate the oxidized fish oil-induced effects on animals.

The yellow catfish *Pelteobagrus fulvidraco*, an omnivorous freshwater economic fish, is widely cultured in several Asian countries, including China, due to its good fillet quality and high economic value [16]. Yellow catfish was chosen as the model fish because its genome is already known. Moreover, under the intensive aquaculture, yellow catfish easily stores excessive lipid in the liver and the intestine. In fact, yellow catfish has been used to study metabolic regulatory mechanisms because its metabolic characteristics and mechanisms are similar to those of mammals [17,18,19]. Herein, we explored the effects of the interactions of selenium and oxidized fish oil interactions on the intestinal lipid metabolism and the antioxidant responses of yellow catfish.

## 2. Materials and Methods

### 2.1. Ethic Statement

Our experimental protocols adhered to the Huazhong Agricultural University (HZAU) ethical rules for the care and use of laboratory animals, and they were approved by the HZAU Ethics Committee.

### 2.2. Expt 1: In Vivo Study

#### 2.2.1. Animals Feeding, Management, and Sampling

The protocols for feed formulation, yellow catfish farming, and their management were similar to those which were described in our recent study [19]. Four diets were produced, including the diets containing an adequate amount of selenium (0.25 mg kg^−1^) with 3% fresh fish oil (A-Se+FFO), an adequate amount of selenium with 3% oxidized fish oil (A-Se+OFO), a high amount of selenium (0.50 mg kg^−1^) with 3% fresh fish oil (H-Se+FFO), and a high amount of selenium with 3% oxidized fish oil (H-Se+OFO) (Supplemental Table S1). An adequate amount of selenium was thought to be that which meets the dietary selenium requirements of yellow catfish, as based on the study by Hu et al. [20]. The fresh fish oil and oxidized fish oil were added the diets, as based on our recent publication [7]. Fresh fish oils were oxidized by heating them to 60 °C, and aerating them for 4 days, and the degree of oxidation was assessed by determining their peroxide value (POV) [21]. The final POV in the four diets was 3.85, 89.69, 3.57, and 86.58 meq kg^−1^ for A-Se+FFO, A-Se+OFO, H-Se+FFO, and H-Se+OFO, respectively. The final selenium contents in the four diets were 0.24, 0.25, 0.49, and 0.48 mg kg^−1^ for A-Se+FFO, A-Se+OFO, H-Se+FFO, and H-Se+OFO, respectively. All of these experimental diets were stored at −20 °C freezer until the feeding procedure was conducted.

The feeding experiment was conducted in indoor fiberglass tanks, and each tank contained 300-L water. Three hundred and sixty yellow catfish (body weight: 3.99 ± 0.01 g/fish, mean ± SEM) were stocked in 12 tanks, with 30 fish/tank. Each type of diet was used to feed the fish in three tanks. The yellow catfish were fed to satiation twice daily. The feeding continued for 10 weeks. After 10 weeks of feeding, before the sampling, all of these experimental fish were fasted for 24 h and euthanized with MS-222 solution at 100 mg/L (MilliporeSigma, St. Louis, MO, USA). Then, three randomly selected fish from each tank were dissected to obtain their intestinal samples (anterior intestine, same below), which were rapidly frozen in liquid nitrogen and stored in a −80 °C refrigerator for a subsequent RNA isolation. For the histological observation, the intestinal samples were collected from another three randomly selected yellow catfish and fixed in a tissue fixative. To analyze the intestinal enzymatic activities, the TG and Se contents of another six fish from each tank were randomly selected and their intestinal samples were taken. They were rapidly placed in liquid nitrogen and stored in a −80 °C freezer.

#### 2.2.2. Analysis of Intestine TG and Selenium Contents

The intestinal TG content was detected by using a commercial kit (A110-1-1; Nanjing Jiancheng Bioengineering Institute, Nanjing, China). The total selenium content was determined based on our previous studies [22]. Briefly, 2 mL of 70% HNO_3_ and 1 mL of 35% H_2_O_2_ were used to digest the intestinal and feed samples, respectively, within a closed vessel heating block system (Mars 5, CEM). The solution was used to analyze the selenium concentration according to the standard reference of Se, using a hybrid generation-atomic fluorescence spectrometer (AFS-8530; Haiguang Instruments).

#### 2.2.3. Enzymatic Activity Assays

CPT1 activity was detected as previously described [23]. One unit of enzyme activity was defined as the amount of enzymes that can convert 1 μmol substrate to product at 28 °C per minute, and this was expressed as units per mg soluble protein.

The kits for the detection of CAT (A007-1-1), T-SOD (A001-1-1), T-AOC (A015-1-2), TRXR (A119-1-1), and the soluble protein concentration (A045-2-1) were purchased from Nanjing Jiancheng Institute of Biological Engineering (Nanjing, China). The kits for the determination of the MDA (S0131S), GPx (S0058), and GSH (S0053) contents were purchased from Beyotime Biotechnology (Shanghai, China).

#### 2.2.4. Hematoxylin-Eosin (H and E) of Intestine

The histological observation of the intestine was performed by light microscopy according to our previous reports [23]. Specifically, the intestinal samples were dehydrated in gradient ethanol, embedded in the paraffin, and finally, they were stained with hematoxylin-eosin (H and E).

#### 2.2.5. Quantitative Real-Time PCR (qPCR) Assay

The Trizol reagent (15596026, Thermo Fisher Scientific, Waltham, MA, USA) was used to isolate the total RNA. A qPCR analysis was performed after our previous publications [24]. The primer sequences are given in Supplemental Table S2. Eight candidate housekeeping genes, including β-actin, 18S ribosomal RNA (18s rRNA), b-2-microglobulin (*b2m*), ubiquitin-conjugating enzyme (*ubce*), hypoxanthine-guanine phosphoribosyltransferase (*hprt*), glyceraldehyde-3-phosphate dehydrogenase (*gapdh*), translation elongation factor (*elfα*), and tubulin alpha chain (*tuba*), were tested for their transcription stabilities. Finally, the two most stable genes were selected as the controls using the geNorm (https://genorm.cmgg.be/. Accessed on 24 November 2021). The 2^−ΔΔCt^ method was adopted to calculate the relative mRNA abundances of genes as described previously [18].

### 2.3. Expt 2: In Vitro Study

#### 2.3.1. Cell Culture and MTT Assay

The human embryonic kidney 293T cell lines (HEK293T cells) were cultured and the MTT method was used to test the cell viability [25].

#### 2.3.2. Preparation of Oxidized EPA (oxEPA)

EPA is abundant in fish oils, but it is prone to lipid oxidation. Oxidized EPA was derived from purified and fresh EPA according to earlier studies [26]. By detecting the thiobarbituric acid reactive substances, the MDA contents were analyzed, and the values were 0.18 μM and 1.49 μM for 100 μM fresh EPA and oxidized EPA (oxEPA), respectively. The EPA or oxEPA was diluted to the desired concentration using 10% fetal bovine serum (FBS) before performing the experiment. To ensure that the viability of the HEK293T cells was not affected during the in vitro experiments, the final EPA and oxEPA concentration were 100 μM (Supplemental Figure S3).

#### 2.3.3. Dual-Luciferase Reporter Assay in HEK293T Cells

First, we determined the 5′ cDNA sequence and transcription start site (TSS) of the gene *slc7a11* based on the reported genome sequence of yellow catfish in GenBank databases. The *slc7a11* promoter was cloned according to our previous methods [27]. Then, we constructed the *slc7a11* promoter into a pGL3 basic vector using the ClonExpress™ II One Step Cloning Kit (Vazyme, Piscataway, NJ, USA). The activating transcription factor 3 (ATF3) binding site (5′-GCTGACGTCATC-3′) on the *slc7a11* promoter was predicted using the JASPAR database (http://jaspar.genereg.net/. Accessed on 24 April 2022). The site mutagenesis of the ATF3 binding site was performed with the QuickChange II Site-Directed Mutagenesis Kit (Vazyme, Nanjing, Jiangsu, China). The specific primers for the plasmid construction and mutagenesis are shown in Supplemental Table S3. The different plasmids were transfected in the HEK293T cell line by using Lipofectamine 2000 (Invitrogen, Carlsbad, CA, USA). The relative luciferase activities were measured using the dual luciferase reporter system (Promega, Minneapolis, MN, USA).

#### 2.3.4. Electrophoretic Mobility Shift Assay (EMSA)

We determined the functional binding sites of ATF3 to the *slc7a11* promoter by EMSA based on our previous publication [27]. The nuclear protein isolation in the HEK293T cells was obtained using the kits from Viagene Biotech (China). The nuclear protein content was determined by the bicarbonic acid (BCA) method. Each oligonucleotide duplex in the ATF3 binding site was incubated with 10 μg nuclear extract according to the LightShift Chemiluminescent EMSA Kit (Invitrogen, Carlsbad, CA, USA). We first used a 10 min period for the reaction of the unlabeled probe, and then we spent 30 min incubating the added biotin-labeled probe. The detection was performed by electrophoresis on the 6% native polyacrylamide gels. Here, 200-fold unlabeled oligonucleotide duplexes were used for the competitive analysis with or without mutations. The oligonucleotide sequences for the EMSA are listed in Supplemental Table S3.

#### 2.3.5. Western Blot Analysis

A Western blot was used to analyze the protein expression according to the protocols described in our study [19]. The antibodies included anti-HA (#5017, Cell Signaling Technology, MA, USA) and anti-GAPDH (#2118, Cell Signaling Technology, Danvers, MA, USA). The Vilber Fusion FX6 Spectra imaging system (Vilber Lourmat) and the Image-Pro Plus 6.0 were used to visualize and quantify the protein bands, respectively.

### 2.4. Statistical Analysis

The analysis of all of the data was performed using the software SPSS 26.0 (IBM, Armonk, NY, USA). These experimental results were shown as mean ± SEM. Prior to a statistical analysis, all of the data were assessed for normality by the Kolmogorov–Smirnov test, and the Bartlett’s test was used to analyze the homogeneity of variance between the treatments. The two-way ANOVA was used to test the variability of the indicators among the four groups. The significant differences between the two treatments were performed via the Student’s *t*-test. The significance level was set at *p* < 0.05.

## 3. Results

### 3.1. High Selenium Alleviated the Oxidized Fish Oil-Induced Damage on the Intestinal Histology

**Figure 1:**
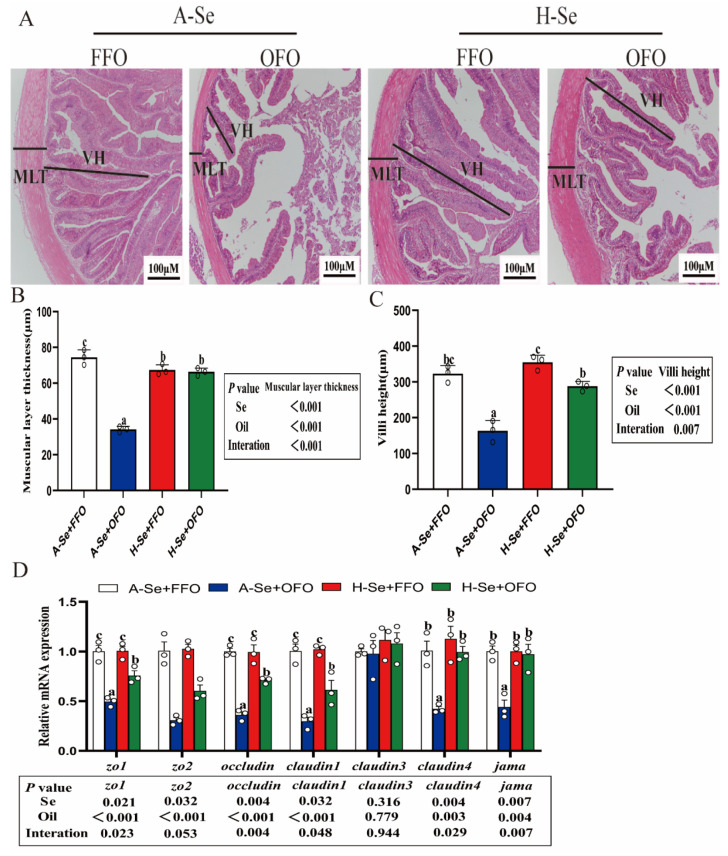
High levels of selenium alleviated the damage that was induced by oxidizing fish oils in the intestine of yellow catfish. (**A**) H and E staining. (**B**) Quantification of intestinal muscular layer thickness. (**C**) Quantification of intestinal villi length. (**D**) The mRNA levels of intestinal barrier. All data were mean ± SEM (*n* = 3). “a–c” indicate meaningfulness at *p* < 0.05. *p* value was calculated by two-way ANOVA. MLT, muscular layer thickness; VH, villi height.

The hematoxylin-eosin (H and E) staining procedure showed that the dietary oxidized fish oils disrupted the normal tissue structure of the intestine, such as it inducing an increased intestinal villi gap and a decreased muscular layer thickness and villi height (Figure 1A–C). The mRNA expression of the genes that are associated with the intestinal barrier were markedly down-regulated by the oxidized fish oils (Figure 1D), including *zo1*, *occludin*, *claudin1*, *claudin4*, and *jama*, and a high level of selenium alleviated the oxidized fish oil-induced damage of the intestinal histology and the decline of these gene expression. Thus, a high level of selenium alleviated the oxidized fish oil-induced damage on the intestinal health of yellow catfish.

### 3.2. High Levels of Selenium Reduced the Oxidized Fish Oil-Induced Lipid Deposition

**Figure 2:**
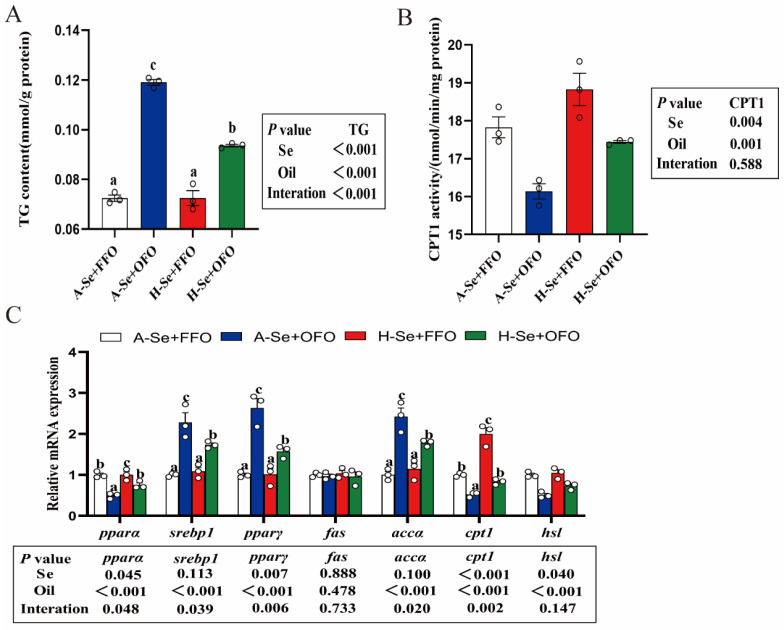
High levels of selenium reduced the oxidized fish oil-induced lipid deposition in the intestine of yellow catfish. (**A**) TG content. (**B**) CPT1 activity. (**C**) The expression of mRNA related to intestinal lipid metabolism. All data were mean ± SEM (*n* = 3). “a–c” indicate meaningfulness at *p* < 0.05. *p* value was calculated by two-way ANOVA. TG, triglyceride; CPT1, carnitine palmitoyl transferase 1.

Next, we investigated the effects of oxidized fish oils and selenium on intestinal lipid deposition and metabolism. Oxidized fish oils enhanced the TG content and induced lipid deposition in the intestine (Figure 2A), inhibited the activity of lipolytic enzyme CPT1 (Figure 2B), and up-regulated the lipogenic genes *srebp1*, *pparγ,* and *accα* mRNA abundance, but they significantly reduced the mRNA abundance of lipolytic genes *pparα*, *cpt1,* and *hsl* (Figure 2C). These results suggested that dietary oxidized fish oils increased the lipid content by enhancing lipogenesis and inhibiting lipolysis. On the other hand, a high level of dietary selenium alleviated the oxidized fish oil-induced increment of the TG content and the mRNA abundances of the lipogenic genes (*srebp1*, *ppar*γ, and *accα*), and the abrogated oxidized fish oil-induced reduction of CPT1 activity and the mRNA expression of lipolytic genes (*pparα* and *cpt1*). Thus, selenium and oxidized fish oils interacted to affect the intestinal lipid metabolism by altering the mRNA abundance and the enzyme activity in both of the intestinal lipogenic and lipolytic pathways.

### 3.3. High Levels of Selenium Attenuated the Oxidative Stress That Was Induced by Oxidized Fish Oils

**Figure 3:**
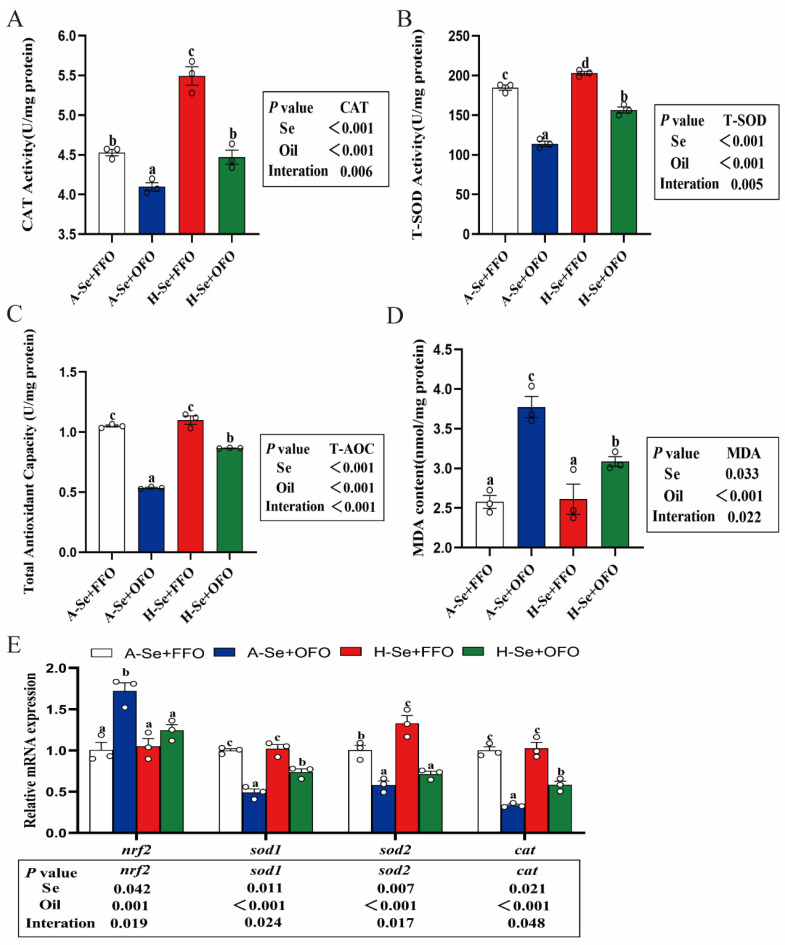
High levels of selenium attenuate the oxidative stress that was induced by oxidized fish oils in the intestine of yellow catfish. (**A**) CAT activity. (**B**) T-SOD activity. (**C**) T-AOC. (**D**) MDA content. (**E**) The genes mRNA expression related to oxidative stress. All data were mean ± SEM (*n* = 3). “a–d” indicate meaningfulness at *p* < 0.05. *p* value was calculated by two-way ANOVA. CAT, catalase; T-SOD, total superoxide dismutase; MDA, malondialdehyde.

The dietary oxidized fish oils markedly reduced the activities of antioxidant enzymes CAT and T-SOD, weakened the total antioxidant capacity (T-AOC), induced oxidative stress, increased the MDA content (Figure 3A–D), up-regulated *nrf2* mRNA expression, but they reduced *sod1*, *sod2*, and *cat* mRNA expression (Figure 3E). These data suggested that oxidized fish oils induced an intestinal oxidative stress. Selenium supplementation significantly alleviated the oxidized fish oil-induced changes to gene mRNA abundance and the enzymatic activities that are relevant to antioxidant responses, indicating that high selenium attenuated the oxidative stress that was induced by oxidized fish oils.

### 3.4. Effects of Selenium and Oxidized Fish Oils on Intestinal Selenoproteins in Yellow Catfish

**Figure 4:**
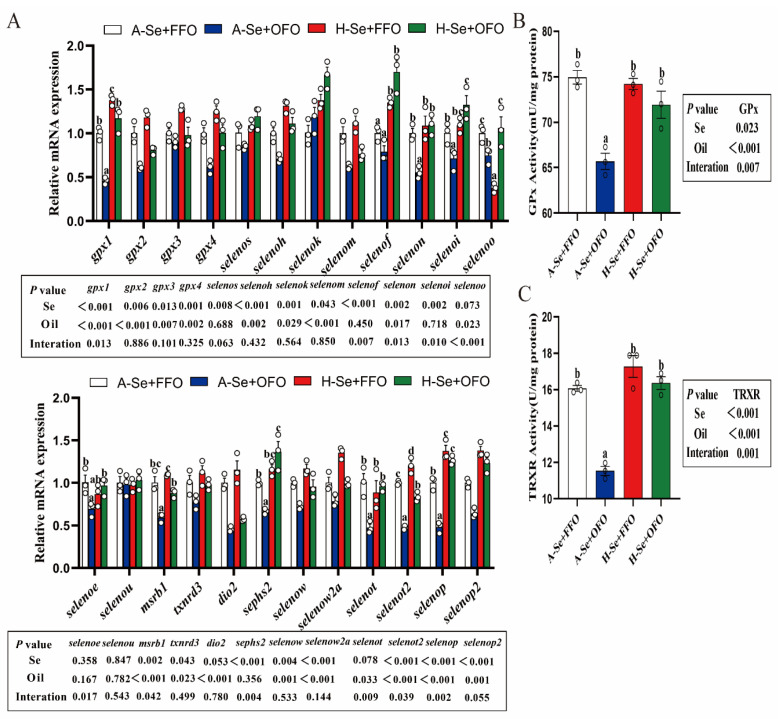
Effect of selenium and oxidized fish oils on selenoprotein expression in the intestine of yellow catfish. (**A**) The mRNA abundance of selenoprotein-related genes in the intestine. (**B**) GPx activity. (**C**) TRXR activity. All data were mean ± SEM (*n* = 3). “a–c” indicate meaningfulness at *p* < 0.05. *p* value was calculated by two-way ANOVA. GPx, glutathione peroxidase; TRXR, thioredoxin reductase.

Selenium and oxidized fish oils interacted to affect the mRNA abundance of several selenoproteins, such as *gpx1*, *selenof*, *selenon*, *selenoi*, *selenoo*, *selenoe*, *msrb1*, *sephs2*, *selenot*, *selenot2* and *selenop* in the intestine. Dietary oxidized fish oils decreased the mRNA abundance of these selenoproteins that are mentioned above, and a high level of selenium alleviated the oxidized fish oil-induced down-regulation of their mRNA abundance (Figure 4A). Dietary oxidized fish oils reduced the GPx and TRXR activities, and a high level of dietary Se addition alleviated these changes which were induced by oxidized fish oils (Figure 4B,C). Thus, selenium and oxidized fish oils interacted to affect the redox homeostasis in the intestine.

### 3.5. Effects of Selenium and Oxidized Fish Oils on Selenium and GSH Contents and Metabolism in Intestine from Yellow Catfish

**Figure 5:**
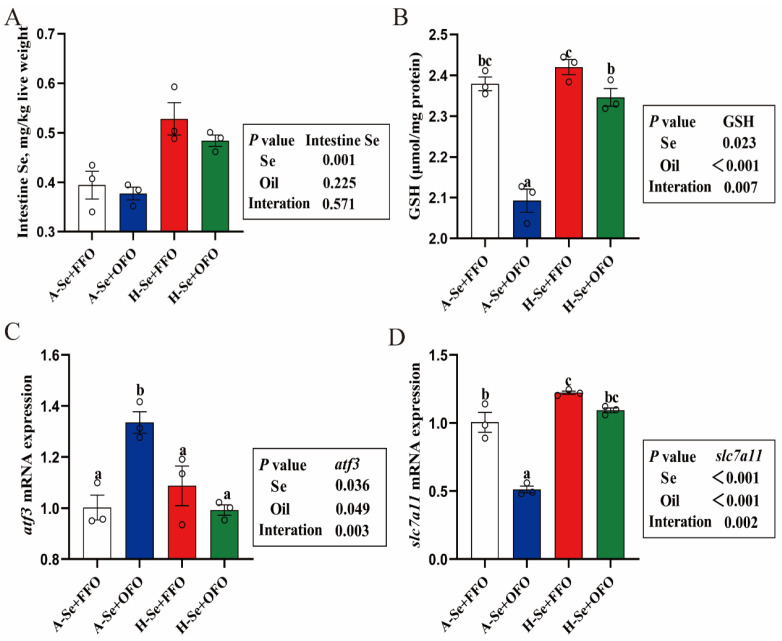
Effect of selenium and oxidized fish oil on Se content and GSH content in the intestine of yellow catfish. (**A**) Se content. (**B**) GSH. (**C**) The expression of *atf3* mRNA. (**D**) The expression of *slc7a11* mRNA. All data were mean ± SEM (*n* = 3). “a–c” indicate significant differences at *p* < 0.05. *p* value was calculated by two-way ANOVA.

Selenium and oxidized fish oils interacted to influence the GSH content, and the mRNA expression of *atf3* and *scl7a11*, but not the selenium content that was in the intestine (Figure 5A–D). Dietary oxidized fish oils reduced the GSH content (Figure 5B) and *slc7a11* mRNA expression, but they increased *atf3* mRNA expression (Figure 5C,D). High levels of dietary selenium alleviated the oxidized fish oil-induced changes to the GSH content and the mRNA abundance of *atf3* and *slc7a11*.

### 3.6. Selenium and oxEPA Affected SLC7A11 Activity by Altering the ATF3 DNA Binding Capacity to the SLC7A11 Promoter in the HEK293T Cells

**Figure 6:**
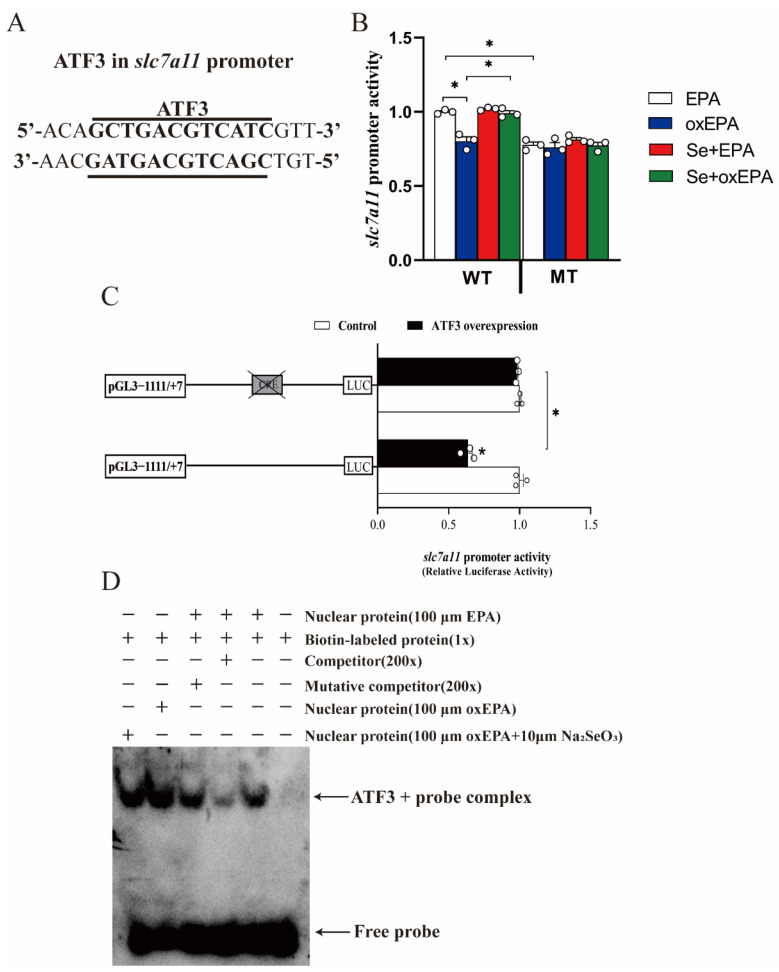
Se and oxEPA affected the SLC7A11 activity by altering the DNA binding capacity of ATF3 to the SLC7A11 promoter in the HEK293T cells. (**A**) Predicted binding site. (**B**) slc7a11 activity assays. (**C**) The luciferase activity assays of predicted ATF3 site after site-directed mutagenesis in the slc7a11 promoter in the HEK293T cells that were treated with an ATF3 overexpression for 24 h. (**D**) EMSA of the predicted binding sequence for slc7a11 promoter for 24 h in the HEK293T cells. All data were mean ± SEM (*n* = 3). “*” indicate significant differences at *p* < 0.05. *p* value was calculated by Student’s *t*-test. ATF3, activating transcription factor 3. EPA, eicosapentaenoic acid; MT, mutant type; oxEPA, oxidized eicosapentaenoic acid; SLC7A11, recombinant Solute Carrier Family 7, Member 11; WT, wild type.

Wang et al. [28] pointed out that ATF3 reduced the SLC7A11 promoter activity. Accordingly, we hypothesized that ATF3 mediated the selenium and oxEPA-induced changes to the SLC7A11 activity. First, we predicted that there could be a ATF3 binding site at -1009/-997 bp of the *slc7a11* promoter of yellow catfish (Figure 6A). Next, we explored the effects of selenium and oxEPA on the *slc7a11* promoter activities. We determined the optimal selenium and oxidized EPA concentrations based on the analysis of the viability of the HEK293T cells (Supplemental Figure S3). Compared to the EPA, the oxEPA significantly reduced the luciferase activity of the *slc7a11* promoter, but Se alleviated the oxEPA-induced decline of *slc7a11* promoter activity (Figure 6B). The mutations in the luciferase reporter reduced the *slc7a11* promoter activity and eliminated the effect of selenium and oxidized fish oil on the *slc7a11* promoter (Figure 6B). The overexpression of ATF3 decreased the luciferase activity of the *slc7a11* promoter compared to the pcDNA3.1 group, and the mutations of the luciferase reporter relieved the ATF3-induced decrease of the *slc7a11* promoter activity (Figure 6C). EMSA showed that the nuclear extracts bind directly to the ATF3 binding sequence that is located on the *slc7a11* promoter. This binding could be inhibited by untagged wild-type probes, but it is reverted by mutant probes (Figure 6D). Furthermore, the oxEPA increased the binding activity of ATF3 to the *slc7a11* promoter, but Se+oxEPA attenuated this binding activity. Thus, ATF3 mediates the interaction of Se and oxEPA to affect the *slc7a11* transcriptional activity.

## 4. Discussion

Our study aimed to investigate the effects of dietary selenium and oxidized fish oils on intestinal lipid metabolism and antioxidant responses. Our main results include: (1) Oxidized fish oils disrupted the intestinal tissues and down-regulated the gene expression of the intestinal barrier, while a high level of dietary selenium could mitigate this negative effect. (2) Oxidized fish oils increased intestinal lipogenesis and inhibited lipolysis, resulting in an increased intestinal lipid deposition; high levels of dietary Se additionally alleviated the oxidized fish oil-induced changes to the lipid metabolism. (3) Oxidized fish oils induced the intestinal oxidative stress and the lipid peroxidation, and a high level of dietary selenium alleviated the oxidized fish oil-induced intestinal oxidative stress. (4) Oxidized fish oils up-regulated the *atf3* mRNA expression, but they reduced the *slc7a11* mRNA expression, and a high level of selenium reversed the oxidized fish oil-induced changes to *the atf3* and *slc7a11* mRNA expression. (5) oxEPA increased the binding activity of ATF3 to the *slc7a11* promoter, but Se+oxEPA attenuated this binding activity.

Fish oils are often added to feeds to help supplement unsaturated fatty acids in fish [29]. However, fish oils are highly susceptible to oxidation, which produces lipid peroxides and poses potentially harmful effects on fish [2], as is observed in our study. The intestine is the site of nutrient digestion and absorption. Studies suggested that the increment of the thickness of the muscle layer and the villi length promote the digestion and absorption capacity of the intestine [30]. Thus, we speculated that oxidized fish oils would reduce the nutrient digestion and absorption by reducing the muscle layer thickness and the villi height, which would in turn reduce the weight gain in OFO-supplemented groups (Zhang et al., under review). This phenomenon has been observed in other studies [31]. Furthermore, our results found that some genes that are associated with an intestinal barrier were significantly down-regulated by the supplementation of oxidized fish oils, which is, again, in agreement with other study [4]. Studies have suggested that tight junction proteins, such as zonula occludins, *occludin*, claudins, and jama, are closely linked to the structural integrity of the intestine and their upregulation can support the normal structure of the intestine [32,33]. Defects in intestinal barrier function increases the probability of intestinal disease [34]. Our study indicated that dietary Se addition alleviated the oxidized fish oil-induced damage of intestinal histology and alleviated the oxidized fish oil-induced down-regulation of the gene expression of the intestinal physical barrier, suggesting that there are beneficial effects of dietary selenium addition in oxidized oils-based diets. Other studies showed that dietary selenium addition could maintain the structural integrity of the intestine by increasing the expression of tight-junction proteins [35].

Several studies have suggested that dietary oxidized fish oils disrupted the lipid metabolism of aquatic animals [5,7]. In this study, oxidized fish oils increased the TG content and inhibited the CPT1 activity. Similar results have been described in other studies [4,5,36]. Our further results revealed that oxidized fish oils up-regulated the mRNA abundance of the lipogenic genes and down-regulated the lipolytic genes, and that adequate amounts of selenium did not alleviate the lipid deposition that was caused by oxidized fish oils. Similarly, Zhang et al. [7] reported that the oxidized fish oils increased the hepatic lipid content by inducing lipogenesis and reducing lipolysis. Selenium plays a wide role in the regulation of lipid metabolism [22]. However, the detailed regulatory mechanisms of this are not yet clear. Oxidative stress is one of the triggers of many diseases [37]. Many studies have confirmed the important role of oxidative stress in the induction of lipid deposition [38,39]. Thus, by considering the high antioxidant capacity of selenium, we speculated that a high level of selenium would regulate the homeostasis of lipid metabolism by attenuating the oxidative stress that was induced by oxidized fish oils.

The long-term intake of oxidized fish oils triggers the oxidative stress in many aquatic animals [3,40]. CAT, GPx, TRXR, and T-SOD are important antioxidant enzymes, and these can remove excess intracellular ROS [37]. We found that their activities were significantly reduced, but MDA, the product of lipid peroxidation, was increased by the use of oxidized fish oils, indicating the activation of oxidative stress. In addition, we found that the activity of CAT and T-SOD paralleled the changes with their mRNA expression, indicating that their activities were controlled at the transcriptional level. Antioxidant enzymes are considered to be important indicators to evaluate antioxidant capacity [19]. Our data showed that oxidized fish oils induced the imbalance in the redox homeostasis by impairing the expression of antioxidant enzymes in the intestine. Similar results were reported by other studies [3,4,5,6,7,31,40]. Nuclear factor E2-related factor 2 (Nrf2) is an essential antioxidant transcription factor that is activated by oxidative stress and helps to increase the antioxidant defense of cells [41]. In our study, the *nrf2* mRNA expression was significantly upregulated in response to the oxidative stress that was induced by oxidized fish oils, which is similar to the results of other studies [39]. Selenium and selenoproteins are essential players in the antioxidant defense of organisms. Our findings showed that high levels of selenium alleviated the oxidative stress that was induced by oxidized fish oils. Moreover, for the first time, we found that the mRNA expression of some selenoproteins was affected by the interactions between selenium and oxidized fish oils, including *gpx1*, *selenof*, *selenon*, *selenoi*, *selenoo*, *selenoe*, *msrb1*, *sephs2*, *selenot*, *selenot2* and *selenop*. Studies have suggested that *gpx1* can be used as the biomarker under various stress conditions [42,43]. We found that oxidized fish oil reduced the mRNA expression of *gpx1*, which might result from the depletion of gpx1 during the antioxidant process, as was suggested by Shi et al. [4]. Our study indicated that H-Se+FFO and H-Se+OFO increased the mRNA expression of *gpx1*. Other studies have suggested that gpx1 had a lower selenoprotein hierarchy and was susceptible to high selenium levels [44].

The GSH is the most abundant antioxidant in cells and its function is to remove ROS [45]. Our results showed that oxidized fish oils reduced the GSH in the intestine of yellow catfish, but a high level of dietary selenium alleviated the oxidized fish oil-induced reduction of GSH, suggesting that a high level of selenium enhanced the intestinal antioxidant capacity. Similarly, studies have reported that oxidized fish oils decreased the GSH contents in several fish species [4]. GSH synthesis depends on the amino acid antiporter system Xc^−^, which is associated with the exchange of extracellular cystine and intracellular glutamate across the plasma membrane. SLC7A11 encodes the light chain, which is a subunit that is specific to the system Xc^−^, and the SLC7A11 expression is usually positively linked to the activity of the antiporter [46]. ATF3 is a stress-inducible transcription factor [47]. Wang et al. [28] suggested that ATF3 binds to the promoter of the SLC7A11 gene to downregulate the expression level of SLC7A11. Our results revealed that a high level of selenium alleviated the oxidized fish oil-induced reduction of the GSH content and *slc7a11* expression and alleviated the oxidized fish oil-induced increase of *atf3* mRNA expression. Studies have suggested that oxidative stress increased the ATF3 expression [47], and that selenite up-regulated the expression of SLC7A11 [48]. Thus, it is reasonable to speculate that ATF3 mediated the alteration of *slc7a11* mRNA expression by the use of selenium and oxidized fish oils, thus, this affected the intestinal GSH content. In our study, we predicted the presence of ATF3 binding sites at the *slc7a11* promoter in yellow catfish. For the first time, we found that ATF3 mediated the effects of Se and oxEPA on the *slc7a11* promoter. Se+oxEPA attenuated the increase of the binding activity of ATF3 to the *slc7a11* promoter.

## 5. Conclusions

In summary, our findings have suggested that a high level of dietary selenium alleviates the negative effects that are caused by oxidized fish oils in yellow catfish, including intestinal structural damage, the disruption of lipid metabolism, and the induction of oxidative stress. We also elucidated the novel mechanism of selenium increasing the GSH content by affecting the interaction of the ATF3 and the *slc7a11* promoter. The results of this study help us to better understand the effects and the mechanism of dietary selenium and oxidized fish oil addition to fish diets on the physiology and metabolic processes of aquatic animals.

## Data Availability

The data presented in this study are available on reasonable request from the corresponding author.

## Supplementary Materials

The following are available online at https://www.mdpi.com/article/10.3390/antiox11101904/s1, Figure S1: Nucleotide sequence of yellow catfish *slc7a11* promoter. The highlighted sequences denote binding sites for putative transcription factors (Expt. 2), Figure S2: The protein expression of *atf3* for 24 h overexpression in HEK293T cells (Expt. 2), Figure S3: Cell viability of the HEK293T cell. (A) Cell viability of the HEK293T cell incubated with EPA or oxEPA-containing medium for 24 h. (B) SLC7A11 promoter activity in the HEK293T cell incubated with EPA or oxEPA-containing medium for 24 h. (C) Cell viability of the HEK293T cell incubated with EPA or oxEPA-containing medium for 24 h with or without Se treatment. (D) SLC7A11 promoter activity in the HEK293T cell incubated with EPA or oxEPA-containing medium for 24 h with or without Se treatment (Expt. 2). Table S1: Feed formulation and proximate analysis of experimental diets (Expt. 1), Table S2: Primers used for quantitative real-time PCR analysis (Expt. 1), Table S3: Primers used for the analysis of the regions of *slc7a11* promoter (Expt. 2).

## Abbreviations

ACC: acetyl-CoA carboxylase; ATF3, activating transcription factor 3; CAT, catalase; CPT1, carnitine palmitoyl transferase 1; EMSA, Electrophoretic mobility-shift assay; GSH, glutathione; GPx, glutathionine peroxidsae; HSL, hormone-sensitive lipase; MDA, malondialdehyde; MLT, muscular layer thickness; Nrf2, Nuclear factor E2-related factor 2; PPAR, peroxisome proliferator activated receptor; ROS, reactive oxygen species; SELENO, selenoprotein; SLC7A11, recombinant Solute Carrier Family 7, Member 11; SREBP, sterol regulatory element-binding protein; TG, triglyceride; T-SOD, total superoxide dismutase; TSS, transcription start site; TXNRD, thioredoxin reductase; UTR, untranslated region; VH, villi height.
