# Supplementary material for: Effects of Dietary Selenium and Oxidized Fish Oils on Intestinal Lipid Metabolism and Antioxidant Responses of Yellow Catfish Pelteobagrus fulvidraco
Source: Antioxidants (Basel). 2022 Sep 26;11(10):1904. doi: 10.3390/antiox11101904 (PMC9598306; doi:10.3390/antiox11101904)
Supplement: Supplementary file 1 [file antioxidants-11-01904-s001.zip › antioxidants-1863824-supplementary.pdf]

```
-1119 GTCTCTGGTT ACTACATATT ACAGACTGCA CACAGTACAA ATGACGTCTT GAAAGTTTTC CACAGGCACT CCTTGAAGAT TAGATCCTGG ACCCTGTGTA
-1019 CAGCTGACGT CATCGTTCAT CATATCAGGG TTTATTTTGC TCTTACCAGT TTTCTGCTTG TTTGCCAGAA ATTTTGATAT TTTTGTGTAG TCACCATGAC
           ATF3
-919  TGGAGCTGAC TCTTGTGAAA GCTCAAATGA GTGTCGTAGA CTGTCTCAGT ATCACACGTC GGTCGTGGGT GGAGCCGTTA ATGATGTAAC GTGATGTTGC
-819  ATCATTTTTC ATTTTTTTTT TCTTCATGCC TTCTTCAGAA ATGTTTTGTT CAAAGACACA CTAACGTAAG AGGCCATAGC TTTAGAGAAA TTAGCTATGT
-719  GGTTGAATTA TGAATTATAT CCTTTATAAC TTCTAAACTT ATACAGTAAA CATGTTATAA TAACGTATTG TGCTTTACGT GTATATTTAA GTACTGGGTC
-619  TAACACATTG TTTCTATGGT AACAGCTCAT TCACGGTAAC TAGTATATTA GCATATGCAA ATAAGAATAA AACAATAAAT CTTATACAAA CATCTTATTC
-519  AATAAAGACG ACGTCTAAGA TGAAGCTTTT TTTTAAGGAC ACAGACAGCT ACAGAACATT CATGGAAGGT TCAGAAATTT CCGGTTTCCT GTTTTTTTGT
-419  TTGTTTGTTT GTTTGTTTTG GGGGTTTTTT TTGGGTTTAT TTATTGAATT TTTTGGGTTA TATAATTTTA AGTTCTCATG TTATGAATTA TTTATGAATA
-319  AGGGCTTCAT CGGAAGTGAA ATGACTAAAG AAATAATGAT TTGATTCCAC GGTTCATGCT AAATCAGCCT CAAACTGGTC TTTTAAAAAA AAAGAAAAGA
-219  AAAAAAAAGA AAGAAAGAAA AAAGAAAAAG AAAATAAAAA GAATTTCAGG CTCACAGTCA AATCAAGAGC TTTGTAGTCA GGGCACGTGT GTGAGCAGTG
-119  AGTTGGAGAT TTGTTAGAGT GAGACAGACA GTGAGAGAGA GAGAGAGAGA GAGAGAGAGA GAGAGAGAGA GAGAGAGAGA GAGAGAGAGA GAGAGATGTA
-19   AGCTAATAGA GAGCAGGGG
            TSS
```

Supplemental Figure S1 Nucleotide sequence of yellow catfish *slc7a11* promoter. The highlighted sequences denote binding sites for putative transcription factors. TSS, transcription start site.

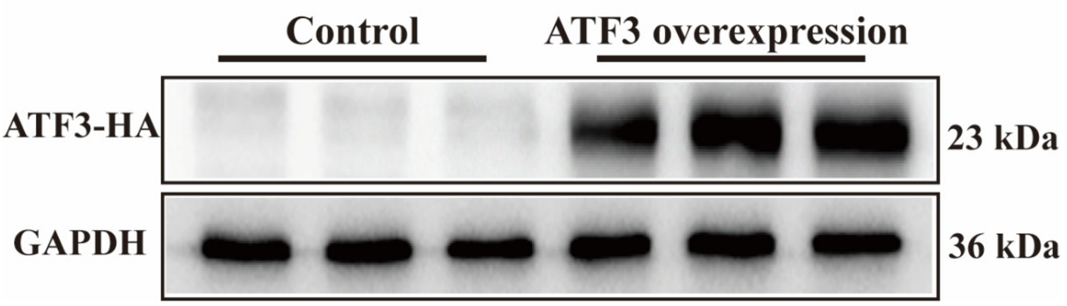

Supplemental Figure S2 The protein expression of *atf3* for 24 h overexpression in HEK293T cells.

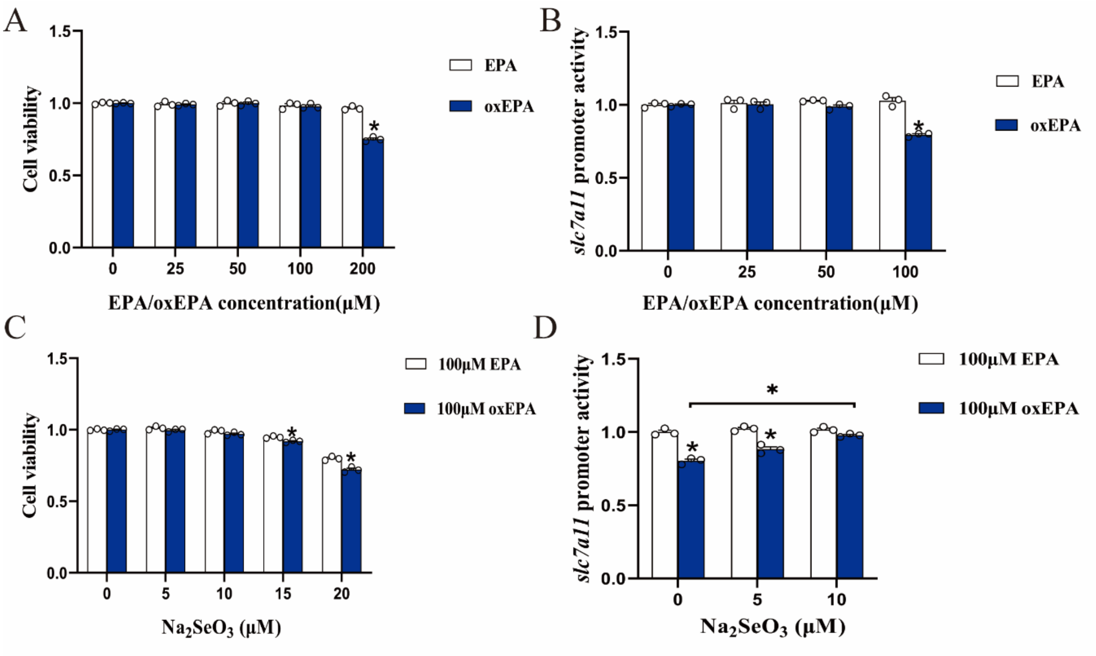

Supplemental Figure S3 Cell viability of the HEK293T cell. (A) Cell viability of the HEK293T cell incubated with EPA or oxEPA-containing medium for 24 h. (B) SLC7A11 promoter activity in the HEK293T cell incubated with EPA or oxEPA-containing medium for 24 h. (C) Cell viability of the HEK293T cell incubated with EPA or oxEPA-containing medium for 24 h with or without Se treatment. (D) SLC7A11 promoter activity in the HEK293T cell incubated with EPA or oxEPA-containing medium for 24 h with or without Se treatment. **P*< 0.05 (Student's t test). EPA, eicosapentaenoic acid. oxEPA, oxidized eicosapentaenoic acid. Se, Na_2SeO_3.

Supplemental Table S1 Feed formulation and proximate analysis of experimental diets

	A-Se+FFO	A-Se+OFO	H-Se+FFO	H-Se+OFO
Ingredients (g/kg)				
Casein	360	360	360	360
Gelatin	10	10	10	10
Corn gluten meal	50	50	50	50
Fish meal	100	100	100	100
Fresh fish oil	30	0	30	0
Oxidized fish oil	0	30	0	30
Corn oil	30	30	30	30
Wheat flour	250	250	250	250
Ascorbyl-2-polyphosphate	10	10	10	10
NaCl	10	10	10	10
Ca(H2PO4)2·H2O	10	10	10	10
Vitamin premix1	5	5	5	5
Mineral premix2	5	5	5	5
Betaine	10	10	10	10
Na2SeO3	0.00037	0.00037	0.00092	0.00092
Cellulose	119.9996	119.9996	119.9990	119.9990
Proximate analysis				
Moisture	79.1	78.9	79.4	79.5
Crude protein	400.8	401.2	401.3	401.5
Crude lipid	98.2	98.4	98.7	98.5
Ash	49.6	49.8	49.3	49.5
Se content	0.00024	0.00025	0.00049	0.00048
Peroxide value (meq/kg)	3.85	89.69	3.57	86.58

Note: 1. Vitamin premix mg provided/kg diet: retinyl acetate 3; cholecalciferol 0.025; dl-α-tocopheryl acetate 30; menadione nicotinamide bisulfite 7; thiamine hydrochloride 6; riboflavin 3; pyridoxine hydrochloride 12; D-calcium pantothenate 30; niacin 50; biotin 1; folic acid 6; cyanocobalamine 0.03. 2. Trace mineral premix mg provided/kg diet: $Ca(H_2PO_3)_2 \cdot H_2O$, 1000; $FeSO_4 \cdot 7H_2O$ 40; $ZnSO_4 \cdot H_2O$ 100; $MnSO_4 \cdot H_2O$ 40; $CuSO_4 \cdot 5H_2O$ 2; $CaIO_3 \cdot 6H_2O$ 3.

Supplemental Table S2 Primers used for quantitative real-time PCR analysis

Genes	Forward primer (5'-3')	Reverse primer (5'-3')	Accession no.
selenow2a	TACGAACCCCGCTATCAGGA	ATCGTGGGCTTTCTGGATGG	ON109398
selenop2	CAGGACGCTTTTTCAAGGGC	GGCAGCACAATGTGAAACGT	ON313812
selenot2	CACACACAGGCAGAACAAGC	AGCTGGCTGATAGACCTGGA	ON109397
gpx1	ACTACACTCAGATGAACGAGCTC	CAAAGTTCCACGAGACGTCATTC	MN062284
gpx2	TCCCCTGTAATCAGTTTGGCTAC	GCCTTTGAATGTCGGGTTCTATG	XM_027172340.1
gpx3	AGAACAACATCTGGGTCTCTGTC	GATTTTCCCCAGGTTCCTGTTTC	MN062285
gpx4	CATCATCACGAACGTAGCCTCTA	CCATTGGTCCGTATCTCTTCACA	MN062286
selenof	GGCTTTTACCGTTGCTTCAGT	ACCTCCCCAATTTTCATCCA	XM_027150758.1
selenok	ACAGTAGGACACAGTCGCCA	GCTTGACGAGGGTCTGAAAGA	XM_027166810.1
selenom	GCTGCGTTTCTTCCATGCTT	TCCTCCACAACTCTCCACCT	XM_027145607.1
selenon	CCGCATCTGGGCTTTATTC	GCGACGCCTGTGAGTTTCT	XM_027143731.1
selenot	CCCTCTGCCTATTTACCGGC	CCAAACAGCATGAACGGGTC	XM_027156829.1
selenoh	GCGTGAGGCTCTCTTCTCTG	TTTACGGGGAGGACCCTTCT	XM_027154592.1
selenos	TCCGTGGTAATGCGTCAGG	TTTGTCCGTCTTGGGCTTC	XM_027163809.1
selenow	GTACAGGCCCAAGTTCACCA	TCCCCGTTCTTCTTCGAGTG	MN062288
selenop	TGTGGAAGGTCGGTGATGTG	TTGCTAAGCCTGCATCCTCC	XM_027171023.1
sephs2	GTCCCTGATCCAGACTACAGATTT	TGTCACAGAAGTGCCTCCCTC	XM_027170772.1
txnrd3	AGACAAGGCTGGGGTGATTG	GACCGCAGCTACCATACTCC	MN062291
dio2	TCTCTCTGGAAGTCGCCTGA	CGAAGTGCAATGCTCGGTTT	XM_027157036.1
msrb1	TCTGAATGACGGACCCAAGC	ACTCTACTGTCCATCTGCCTCT	XM_027141750.1
zo1	ACTACGGCATGGAAAACCAG	CCTGGTTTGTAGCCACGATT	XM_027173133
zo2	ACTACGGCATGGAAAACCAG	CCTGGTTTGTAGCCACGATT	XM_027173133
occludin	AGGAGGCTATGGCTCAAACA	CAAGGACAGAATCGCACAGA	XM_027155195
claudin1	CATCAGACGGCAGTTCTTCA	TGCTGGACCTCCTGAACTCT	XM_027176967

claudin3	CATCTGGATGACCTGTGTGG	AATCATGACCTTTGCCTTGG	XM_027148913
claudin4	GGCAGAAGGTTTTTGAGCAG	GTTCCTGCCAGTTCAACCAT	XM_027148477
jama	TCCTCCCTCCACCTATAAATGGT	CCTGTGAAGGCTGTGTATACACT	XM_027165566.2
pparα	AGGCTTCCACTATGGTGTGC	TGGCACTTGTTCCGGTTCTT	KF614122.1
pparγ	ACGCCCCGTTCGTTATCC	TGAGCAGAGTCACCTGGTCATTG	JX992741
srebp1	CTGGGTCATCGCTTCTTTGTG	TCCTTCGTTGGAGCTTTTGTCT	JX992742
fas	AACTAAAGGCTGCTGGTTGCTA	CACCTTCCCGTCACAAACCTC	JN579124
accα	GGGGTTTTCACGCTGCTTC	GGTTCTGATTGGGTCGTCCTG	JX992746
cpt1	ATTTGAAGAAGCACCCAGAGTATGT	CCCTTTTATGGACGGAGACAGA	JQ074177
hsl	GAAGGACAGGACAATGAGAAGC	TGTACCACCAGCCAAGGAGA	KJ588765
sod1	CCTCAAAGGCACAGGAGAAG	AATCGGCAGTCACATTACCC	XM_027171881
sod2	TCATGCAGCTTCACCATAGC	CTGTGGTTCTCCTCCACCAT	XM_027166181
cat	CGTTTAGCCGCTACTGATCC	CTGGAATCAGGGGGTAGTCA	KX455919
nrf2	GTGAAGGGGGAAACACAGA	GCTCGTCCATGTCAGAGTCA	KX455917
slc7a11	CTGACCAACGTCGCATATTACAC	TGTAGAGGTCTCCGAAAAACAGG	XM_027163602
atf3	GAACGCTTACTGTGGAAGAACAC	GTGGTGAAGTTCATACACTGCAG	XM_027138056
18s rRNA	AGCTCGTAGTTGGATCTCGG	CGGGTATTCAGGCGAGTTTG	KP938527
β-actin	GGACTCTGGTGATGGTGTGA	CTGTAGCCTCTCTCGGTCAG	EU161066
gapdh	GCCTCCTGCACCACAAACT	GCCTTGTTGAGCTTGACGAA	KP938522
tuba	TCAAAGCTGGAGTTCTCGGT	AATGGCCTCGTTATCCACCA	KP938526
b2m	GCTGATCTGCCATGTGAGTG	TGTCTGACACTGCAGCTGTA	KP938520
elfa	GTCTGGAGATGCTGCCATTG	AGCCTTCTTCTCAACGCTCT	KU886307
ubce	TCAAGAAGAGCCAGTGGAGG	TAGGGGTAGTCGATGGGGAA	KP938524
hprt	ATGCTTCTGACCTGGAACGT	TTGCGGTTCAGTGCTTTGAT	KP938523

Abbreviations: *accα*, acetyl-CoA carboxylase *α*; *atf3*, activating transcription factor 3; *b2m*, beta-2-microglobulin; *cat*, catalase; *claudin1*,*3*,*4*; claudins 1, 3, 4; *cpt1*, carnitine palmitoyl transferase 1; *dio2*, deiodinase 2; *elfa*, translation elongation factor; *fas*, fatty acid synthase; *gapdh*, glyceraldehyde-3-phosphate dehydrogenase; *gpx*, glutathione peroxidase; *hprt*, hypoxanthine-guanine phosphoribosyltransferase; *hsl*, hormone-sensitive lipase; *jama*, junctional adhesion molecule A; *msrb1*, methionine sulfoxide reductase b1; *nrf2*, nuclear factor E2-related factor 2; *occludin*, occludin; *ppar*,

peroxisome proliferators-activated receptor ; *selenoe, f*, *h*, *i, k*, *m*, *n*, *o*, *p*, *s*, *t*, *u*, *w*, *t2*, *p2*, *w2a*, selenoprotein e, f, h, i, k, m, n, o, p, s, t, u, w, t2, p2, w2a; *sephs2*, selenophosphate synthetase 2; *slc7a11*, recombinant Solute Carrier Family 7 Member 11; *sod1*, *2*, superoxide dismutase 1, 2; *srebp1*, sterol regulatory element binding proteins 1; *tuba*, tubulin alpha chain; *txnrd*, thioredoxin reductase; *ubce*, ubiquitin-conjugating enzyme; *zo1*,2, zonula occludens 1, 2; *18s rRNA*, 18S ribosomal RNA;

Supplemental Table S3 Primers used for the analysis of the regions of *slc7a11* promoter

Primers	Sequences (5'-3')
slc7a11-pGL3-1111/+7-F	ctatcgataggtaccgagctcATATTTAACCCTCTGTCTCTGGTTACTACA
slc7a11-pGL3-1111/+7-R	cagtaccggaatgccaagcttCCCCTGCTCTCTATTAGCTTACATC
ATF3-HA-1-F	GAACAAGCCTGGGGAGAG
ATF3-HA-1-R	CAGCCTGAGAAGGTCAAA
ATF3-HA-2-F	ATGTACCCATACGACGTCCCAGACTACGCTATGCTTCAG
ATF3-HA-2-R	AATACAAGAAAAAGACAT
slc7a11-mut-F	CAttacgatcgcaaGTTCATCATATCAGGGTTTATTTTGC
slc7a11-mut-R	GAACttgcgatcgtaaTGTACACAGGGTCCAGGATCTAATC
slc7a11-Biotin probe-F	ACAGCTGACGTCATCGTT
slc7a11-Biotin probe-R	AACGATGACGTCAGCTGT
ATF3 mut competitor-F	ACATTACGATCGCAAGTT
ATF3 mut competitor-R	AACTTGCGATCGTAATGT
